# Professional Letters from Paris

**Published:** 1852-12

**Authors:** Paul F. Eve

**Affiliations:** Paris


From the Nashville Medical Journal.

*Professional Letters from Paris—Velpeau—Chomel—Hotel Dieu—Nelaton—Civiale--Roux.*

PARIS, May 25, 1852.

*Dear Doctor*—I arrived here safe on the 21st, and soon met our friends, Prof. Lindsley and Drs. Duval, Brackenridge and some others, who, like myself, have been attracted to this head quarters of medical science. I find our colleague labors in the laboratory nine hours a day.

I have only as yet visited La Charite and shaken hands with the great Velpeau. He has but little of much interest in his wards. I saw him divide the tendon of the tibialis anticus muscle and operate for cataract. He has one curious case, to which he specially directed my attention. This is a salivary fistula, situated about three quarters of an inch behind the angle of the inferior maxillary bone, in a young man of 19 years. He says he had an abscess in the parotid region, which was open seven years ago, and this fistula is the result. The peculiarity of this case is in its position, so distant from the canal of Steno.

In his lecture of this morning, he dwelt upon the therapeutic effects of tinct. Iodine in mammary abscesses. Used of full strength, he has known excellent results in abscesses even with free suppuration, provided there is no internal organ affected.

We read this morning in the Presse,—one of the few newspapers which are now permitted to appear in Paris,—that one of the most learned and celebrated professors of the Faculty of Paris, M. Chomel, has resigned, refusing to take the oath to the new government. The chair of internal clinic is thus left vacated in the school of Medicine.

It is said that the Prince President is about to abolish the system of "concours," by which the Professorships have hitherto with so much satisfaction been filled.

The old Hospital, Hotel Dieu, we are informed, is about to be brokeu up or removed. I think you will agree with me that this ought to be done, when we find in one of the guide books of Paris, the assertion made, (I cannot say fact, for I hope for humanity and our profession it is not true,) that of the first 500 patients received into it during the cholera of 1832, only one survived; and only five of the first thousand. The location of this Hospital is certainly very bad.

May 26th.

Heard M. Nelaton lecture this morning, at the Hospital of the School of Medicine. My expectations were fully realized. He is an excellent clinical lecturer. In the course of the morning he used the blackboard three times to illustrate his positions and remarks. He first exhibited the pathological specimen of an old man who had died with an artificial anus. While preparing for an operation, he was attacked with pneumonia and immediately suffered from bed sores about the sacrum and hips. M. Nelaton remarked that M. Malgaigne had first observed, that while patients paralytic, for instance, may be upon the back for months without excoriations, yet the moment an acute inflammation attacks them, bed sores are the result. So in his case; the patient, although aged, was doing well until the lungs became invaded. Another remark he made was, that in all the post mortem examinations he had made of patients who had labored under artificial anus, he had invariably found the upper portion of the intestine involved in the affection inside, and the lower portion outside, as regards the mesial line of the body. His explanation is, that the upper portion of the bowels becomes distended and falls naturally towards the pelvis, while the lower portion being empty, is consequently pushed outwards or to the iliac fossa of one or the other side.

He next alluded to a case just received into the Hospital, seriously injured by a fall. In relation to the question of diagnosis of infiltration of blood under the scalp and fracture of the cranium, he said one could be easily distinguished from the other by these symptoms:

1st. In a bloody tumor simulating a fracture, let the surgeon press steadily upon it, and the fluid being displaced, he will feel the arm resisting the bone.

2d. Should an artery, divided in the injury, give rise to the pulsation, according to its situation, compress the temporal or occipital vessels, and it will cease in the bloody infiltration.

3d. The pulsation of the brain differs from that of an artery; in one case it is an artery, in the other it is a mass moved by several vessels.

4th and lastly. If the brain be injured, and œdema of the eyelids ensue, the infiltration will take place slowly, and first exhibit itself under the conjunctiva. If the contusion be superficial, the eyelids will become puffed up at once; but if deep-seated, then it will appear gradual and be first subconjunctival. This difference in the same condition of these organs, is owing to the resistance of the membrane connecting the cartilages of the eyelids to the surrounding soft parts.

SATURDAY, 29th.

Went to the Hospital Neckar to see the celebrated Dr. Civiale. He is quite an indifferent lecturer, but an inimitable operator with the catheter or lithotrity instruments. He stated in his *lecon* today, that the statistics of 11,000 cases of lithotomy exhibited one death for every 9 infants, 2 deaths for every 9 adults, and 3 deaths for every 9 aged persons. He says he prefers cutting to dilatation, to cure stricture of the urethra. He operated on a case by crushing a fragment of a stone he had broken at a previous sitting, with the instrument now generally if not universally employed, having a beak like a duck, with two branches, one sliding in the other. He is inclined to the opinion, but not definite, that chloroform prevents reaction.

M. NELATON'S CLINIC.—He presented two cases upon which he had operated. The first was an extensive necrosis of the femur, upon its anterior surface, near the knee-joint. An incision through the soft parts was made, and a variety of strong cutting forceps were employed to remove a considerable number of pieces of bone. The patient was a youth, and placed under chloroform during the operation. It was a tedious and somewhat embarrassing one.

The other one was the removal of the little finger with its metacarpal bone, for caries at the wrist-joint. The operation consisted of an incision from the head of the fifth metacarpal bone to its distal extremity, or metacarpo-phalangeal articulation, around which a palmar and dorsal cut was made, so as to pass the knife between the fourth and fifth bones of the hand, when the latter was disarticulated from the os unciforme. The cutting forceps were also used in the wrist-joint. Chloroform was again used, but did not act as favorably in this as in the previous case.

In alluding to the dangers of the operation, M. N. declared that it could not be performed without opening the carpal articulation.

Mr. Costello, the editor of the Surgical Encyclopedia of London was present at this clinic. He is frequently at this Hospital.

JUNE 1st.

At La Charite. M. Velpeau entered to-day upon the interminable question of cancer, preparatory to removing a diseased mamma. The French still hold to the terms of soft and hard cancers. M. V., like all prudent surgeons, is averse to operate upon every scirrhus, and especially upon ulcerations of a decided carcinomatous character. But like others, he does operate in certain cases. The one of to-day he thought wanting in several particulars to make up genuine scirrhus. I was greatly surprised to see him present the pathological specimen of tiba, which he termed encephaloid cancer, taken from a lady of Paris, who had received a fall some three or four weeks before. What would Mr. Stanly of London say to this carcinoma of a bone?

He removed the entire breast with a chain of glands, small, and not extending very high into the axilla, in two and a half minutes. You know he is not an expert or very dexterous operator; using, as he is compelled to do, the middle and not the fore-finger of the right hand, because of an injury to it in youth. Chloroform was used in this case; acted well.

JUNE 2d.

At the Hotel Dieu. And what a change has come over it since I was a student there in 1830 and '31. I went round the wards with M. Joubert de Lamballe, one of the best surgeons and best lecturers in Paris. I saw several interesting cases in his wards, and there is little doubt he is doing as much for French surgery as any one else. In one case of retention of urine for stricture, the bladder was punctured above the pubis, the stricture cured, a large fistula anterior to the testicles covered by a flap slided from the scrotum, and the patient is now nearly well. A case of stone upon which he operated six days previously was also doing well. The operation was the lateral, and performed with a bistoury.

A fractured leg he was treating with a folded sheet, placed transversely over it, so as to compress the limb upon the bed (hard mattress and folded cloths over it) while extension was maintained from the perineum of the fractured side and the ankle by handkerchiefs to the head and foot of the bedstead. A rhinoplastic operation was not very promising.

After the visit to the wards, I went into the amphitheatre, where I had so often heard Dupuytren lecture to some hundred students. I found the old veteran M. Roux in his place, and counted sixteen students and 9 interns around his table—this was his class, all told, and yet he had several operations to perform. M. Roux lectures, if any thing, worse than ever, being now very old; but still he operates with wonderful skill. At his present age, say near 80, I saw him go through every stage of his favorite method for cataract. Having many years ago operated upon 600 cases by the different processes proposed to relieve cataract, he ascertained that extraction had been the most successful. Without the aid of glasses he performed this operation as well as any one. In extirpating the eye, chloroform was administered by an inhaler while the patient was in a sitting position The impression was not good, and the operation badly performed. More than 8 minutes were consumed in its removal, and the patient suffered greatly.

JUNE 4th.

La Charite. M. Velpeau. His lecture to-day would have pleased you greatly. In relating the symptoms of a diseased os tinsæ, he came out against the modern use (abuse) of the speculum. He declared this instrument was never useful in displacements of the womb, or in diagnosing tumors projecting into the vigina. It was necessary, he admitted, in the topical application of medicaments to the os tincæ. Few instruments had been more abused, and it was high time honest physicians should do all they could to arrest the furor among women for this indecent, unnecessary, and injurious examination. I could not but recollect your satisfactory argument on this subject last winter, when scolded at a consultation of old grannies for not using the speculum—viz. : that as the patient in question did not now bear children, she therefore had no womb, and the instrument was not required.

He removed the little finger of the right hand at the metacarpo-phalangeal articulation, for deformity. The patient had had the hand crushed some years ago, and this finger now projected upon its palmar surface. Velpeau remarked that there were two kinds of operation *per complaisance*—1st, simply to gratify the patient as to appearances; the 2d, because the deformity presents or interferes with his business or daily work. The surgeon, of course, is more excusable in operating under the latter circumstances than for the simple gratification, without any useful object in view.

JUNE 5th.

At Hospital Neckar. M. Civiale's service. Witnessed lithotomy by the lateral operation—instrument, the single lithotome cache; patient, boy of seventeen years; time, three minutes and a half; stone, apparently mulberry, size of a pullet's egg. There was rather too much parade before the operation in preparation for it, but it was well done. Civiale did not operate, but asked his right hand man, M. Le Noir, to perform it. Chloroform acted well in the case.

JUNE 7th.

Clinic of the School of Medicine. M. Nelaton. He gave to-day a most excellent lecture on internal intestinal obstructions. The case provoking the remark was this. A man aged 52 was sent to the Hospital by a friend of M. N., who for five days had no fæcal evacuation, great meteorism of the whole abdomen, stercoraceous vomiting, but no fever; pulse 85. Croton oil was given in large doses, and the whole abdomen covered with ice. These means produced immense fæcal evacuations, with immediate relief to the patient. This morning he is very weak, pulse at 120, no appetite, and it is apprehended he will die.

The surgeon took occasion to enlarge on the subject of intestinal obstructions arising from internal causes. These he mentioned were three. 1st. Those arising from substances foreign to the bowels; they may come from without, or originate within; instances cherry stones, etc., and biliary calculi, etc. 2d. Intestinal ulcerations, particularly in tuberculous subjects. 3d. Strictures, especially produced by the appendix vermiformis. Of this latter variety two specimens were exhibited. He of course spoke of these affections independent of hernia.

He lastly alluded to the operation for the relief of these internal strictures. He said surgeons were averse to operate, because they could not tell where the mechanical obstructions existed, nor could it be always relieved by opening the abdomen. He says Dupuytren, in 1718, proposed to establish, under these circumstances, an artificial anus, by opening the intestines above the stricture. M. Laugnier performed this operation in 1838; result unknown; but next year M. Maisonneuve succeeded perfectly.

M. Nelaton has now operated several times, with and without success, and he now thinks that this ought to be considered an established surgical operation.

In the diagnosis of internal intestinal obstructions, the surgeon must be influenced by its sudden production.

Very respectfully, yours,

PAUL F. EVE.

